# Tumor Hypervascularity and hand-foot-skin reaction predict better outcomes in combination treatment of TACE and Sorafenib for intermediate hepatocellular carcinoma

**DOI:** 10.1186/s12885-019-5570-z

**Published:** 2019-04-30

**Authors:** Enxin Wang, Dongdong Xia, Wei Bai, Jie Yuan, Xiaomei Li, Jing Niu, Zhanxin Yin, Jielai Xia, Hongwei Cai, Daiming Fan, Guohong Han, Lei Liu

**Affiliations:** 1 Department of Liver Disease and Digestive Interventional Radiology, Xijing Hospital of Digestive Diseases, Fourth Military Medical University, Xi’an, 710032 China; 2 Department of Medical Statistics, Fourth Military Medical University, Xi’an, 710032 China; 3 Xijing Hospital of Digestive Diseases & State Key Laboratory of Cancer Biology, Fourth Military Medical University, Xi’an, 710032 China; 4 Cell Engineering Research Center and Department of Cell Biology, State Key Laboratory of Cancer Biology, Fourth Military Medical University, 169 Changle Road, Xi’an, 710032 China

**Keywords:** Hepatocellular carcinoma, Transarterial chemoembolization, Sorafenib, Vascularity, Hand-foot-skin reaction

## Abstract

**Background:**

To validate the robust predictive values of tumor vascularity and hand-foot-skin reaction (HFSR) in combination treatment of transarterial chemoembolization (TACE) and sorafenib for patients with intermediate hepatocellular carcinoma (HCC), and then select the potential candidates who would survive best from such treatment.

**Methods:**

A total of 132 treatment-naive patients with intermediate HCC undergoing combination therapy of TACE and sorafenib were recruited between January 2010 and December 2014. The tumor vascularity was defined according to digital subtraction angiography (DSA) and HFSR was assessed by the national cancer institute common terminology criteria for adverse events (NCI-CTCAE). The Mann-Whitney U test was used to assess the correlation between vascularity and radiologic response; time to radiologic progression (TTP) and overall survival (OS) were evaluated using Kaplan-Meier techniques and compared by log-rank test; factors associated with them were evaluated using multivariate Cox regression analysis.

**Results:**

During a median follow up of 17.3 months, it was revealed that hypervascularity and development of ≥2 grade of HFSR within 60 days after sorafenib initiation were favorable predictors for TTP (HR 0.378, *p* < 0.001; HR 0.627, *p* = 0.018) and OS (HR 0.499, *p* = 0.002; HR 0.555, *p* = 0.004). The median TTP and OS for patients with both were 12.2 and 29.1 months, which were better than patients with either of them (6.0 months, HR 1.74, *p* = 0.012; 16.5 months, HR 1.73, *p* = 0.021), as well as those with neither (2.9 months, HR 3.74, *p* < 0.001; 11.9 months, HR 3.17, *p* < 0.001).

**Conclusions:**

Tumor hypervascularity and development of ≥2 grade of HFSR within 60 days were favorable predictive factors for the combination treatment of TACE and sorafenib, with both of which the patients survived longest and might be the potential candidates.

**Electronic supplementary material:**

The online version of this article (10.1186/s12885-019-5570-z) contains supplementary material, which is available to authorized users.

## Background

Hepatocellular carcinoma (HCC) is the sixth most common malignancy in the world with a continued increase of incidence, and the third leading cause of cancer-related deaths globally [1, 2]. However, nearly 20 % of the patients were diagnosed at intermediate stage; and unfortunately, curative treatments, such as resection, liver transplantation or local ablation, might not benefit them [3, 4]. For these patients, transarterial chemoembolization (TACE) was most frequently used as a palliative treatment worldwide [5]; besides, according to Barcelona clinic liver cancer (BCLC) staging system and treatment guidelines, TACE as the standard therapy could improve the survival from 16 months in untreated patients to 19.6 months in general patients and to almost 40 months in well-selected ones [6–8].

Although the efficacy of TACE has been confirmed, the long-term outcomes remain unsatisfactory, probably for the hardness to achieve complete histological necrosis for the lesions treated by TACE alone [8, 9]. In addition, sorafenib has showed significant improvement in overall survival (OS) and time to tumor progression (TTP) for patients with advanced HCC [10, 11]. Therefore, combining TACE with sorafenib might be an effective intervention for covering the shortages and side-effects of the former treatment alone [12, 13]. In spite of the safety of combining TACE and sorafenib in managing patients with intermediate HCC, its superiority to TACE alone still remains inconclusive [14–16]. Although this combination therapy was used in routine clinical practice for the treatment of intermediate HCC, it might not benefit all of these patients [17].

Nevertheless, there is still absence of reliable biomarker for TACE or sorafenib, as well as the combination therapy of them. Vascularity have been reported as an imaging marker for predicting radiological response on TACE and corresponded well with OS and TTP in patients treated by TACE [18–20]. However, its predictive values in combination treatment of TACE plus sorafenib have never been reported previously. Besides, sorafenib related adverse events (AEs) were widely regarded as surrogate markers for disease control and survival in patients with advanced stage of HCC, especially the hand-foot-skin reaction (HFSR) with the most frequent report and highest predictive value according to our previous studies [21–32].

In this study, we sought to investigate how vascularity and HFSR individually and in combination, correlate with TTP and OS to determine their utility as robust predictors, and hence select the potential candidates based on these factors for combination therapy of TACE and sorafenib.

## Methods

### Study population

This retrospective study comprised 447 newly diagnosed HCC patients who were treated with combination therapy of TACE and sorafenib at our center between January 2010 and December 2014, according to the criteria from the European association for the study of liver disease/American association for the study of liver disease [6, 7]. The patients were eligible for treatment if they presented with unresectable HCC, an eastern cooperative oncology group (ECOG) performance status of ≤1, adequate hematologic and renal function. Excluding 183 patients who had macrovascular invasion, 69 patients with extrahepatic spread, and 3 patients with Child-Pugh score of greater than B8, 192 patients remained after that. Considering that short duration of exposure to sorafenib might impact the judgment of adverse events and the efficacy of combination treatment, 47 patients who were treated with sorafenib for less than 8 weeks were also excluded to rule out the potential time-dependent bias [25, 26]. And then 13 patients were additionally excluded for the interval time of > 60 days between the beginning of sorafenib treatment and the first TACE procedure. Finally, present study included 132 treatment naive patients with unresectable HCC treated by combination therapy of TACE and sorafenib with more than 8-week sorafenib administration. The study protocol conformed to the ethical guidelines of the 1975 declaration of Helsinki and was approved by the ethics committee of Xijing Hospital (Xi’an, China). A written informed consent about receiving treatment and providing their clinical data in following studies was given by all patients before receiving combination therapy according to the institutional guidelines.

### Treatment protocol, evaluation of vascularity and adverse events

Before the TACE procedure, digital subtraction angiography (DSA) of the hepatic artery was performed to assess the vascular anatomy and tumor vascularity. Hypervascular lesions were confirmed by two independent radiologists based on these characteristics: (1) tumor staining obviously; (2) vessels dilated and tortuous; (3) venous pooling; (4) “holding ball” sign; (5) clear boundary of the lesion. Otherwise were considered hypovascular. During the operation, tumor-feeding vessels were selected/super-selected whenever possible, and then infused by a mixture of lipiodol (2–20 mL) and doxorubicin (10–50 mg), followed by an embolization with gelatin sponge particles. The infusion continued until a stagnant flow was observed in the feeding vessels. After TACE procedure, lipiodol retention in DSA further verified the hypervascularity of the lesion. Tumor response was evaluated every 4–6 weeks with dynamic liver CT or magnetic resonance (MR) imaging, along with chest CT and/or bone scanning if applicable, according to the modified response evaluation criteria in solid tumors (mRECIST). For patients with residual viable lesions or local and/or distant intrahepatic recurrences over follow-up, on-demand repeated TACE sessions were carried out; and the TACE therapy was discontinued under the condition of liver function deterioration (Child-Pugh score more than 8), performance status worsening (ECOG score more than 2) and disease progression according to imaging assessments. As for the initiation of sorafenib, there were 74 (56.1%) patients initiating sorafenib before first TACE, while the remaining 58 (43.9%) patients received sorafenib after TACE. The median time interval between the initiation of TACE and sorafenib was − 2 (interquartile range [IQR] -3 to 3.25) days. Sorafenib was administered to the patients at a dosage of 400 mg twice daily without any planned interruption during TACE procedure. Despite does modification based on the presence of adverse events, patients were still encouraged to continue the sorafenib treatment if the toxicity was manageable. In this study, the prevention of sorafenib side effects is not applied; however, when the side effects reached severe or affect life quality, the patients would receive relevant treatment. Grade of adverse events were prospectively defined according to the national cancer institute common terminology criteria for adverse events (NCI-CTCAE) version 3.0. According to our previous studies, the development of a HFSR ≥ grade 2 within 60 days after sorafenib initiation as the optimal criterion to best discriminate responders with improved survival [31]; therefore, this study focused on this kind of AE. After the disease progression, combining therapy was used to these patients still in intermediated stage and sorafenib alone was recommended to those progressing to advanced stage.

### Statistical analysis

Baseline characteristics were summarized using descriptive statistics. Overall survival (OS) was defined as the time from initiation of treatment (either TACE or sorafenib) until death or until last follow-up; and time to radiological progression (TTP) was defined as the time to the radiological confirmation of tumor progression or the last imaging assessment. Survival analysis was carried out using Kaplan-Meier method for univariate analysis and compared with the log-rank test. The Mann-Whitney U test was used to compare ordinal and categorical variables. Cox proportional hazard regression was used for multivariate analysis to confirm the predictive value of vascularity and HFSR, as well as the stratification based on them. Statistical analysis was performed using SPSS software, version 17.0 (SPSS, Inc., Chicago, IL), and a two-sided *p* value < 0.05 was considered significant.

## Results

### Patient characteristics

The baseline demographic and clinical characteristics of patients are shown in Table 1. Among the 132 eligible patients, 112 patients (84.8%) were male with a mean age of 53 years; hepatitis B virus (HBV) was the most common underlying cause of liver disease (82.6%). There were 75 patients (56.8%) diagnosed at BCLC B stage, while 47 patients (35.6%) belonged to BCLC C stage only because of ECOG performance status of 1. 124 patients (93.9%) were in the Child-Pugh A class, and 15 patients had ascites. None of the 8 patients who were classified in the Child-Pugh B class had clinically overt jaundice or hepatic encephalopathy. The median diameter of the largest measurable lesion was 7.1 cm (IQR 5.2–9.8), and 69 patients (52.3%) had single lesion. The median number of TACE sessions was 3 (IQR, 1–4), and 24.2, 22.7, and 25.8% of the patients received 1, 2, or 3 TACE treatments, respectively. Eighty-nine patients (67.4%) received sorafenib treatment before TACE and 43 patients (32.6%) began sorafenib after TACE. For sorafenib therapy, the median duration of its administration was 16.5 (IQR 9.5–26.7) months. There were 23 (17.4%) patients with sorafenib dose reductions due to adverse events, and 32 (24.2%) patients with temporary dose interruptions occurred (17 for AEs, 11 for impairments in general condition or liver function and 4 for other non-disease-related reasons). Finally, 36 (27.3%) patients discontinued the sorafenib treatment because of disease progression (*n* = 12), deterioration of their liver function (*n* = 19) or other reasons (*n* = 5). None of the included patients permanently stopped sorafenib therapy owing to adverse events. For the whole cohort, the median TTP and OS reached 7.3 months and 21.4 months, respectively.

**Table 1:** Baseline demographics and clinical characteristics of the patients (*N* = 132)

Characteristics	Number (%) / mean ± S.D. / median [IQR]
Age at start (year)	53 ± 12.1/53 [43–61]
Gender	
men	112 (84.8%)
women	20 (15.2%)
Etiology	
Hepatitis B	109 (82.6%)
Hepatitis C	6 (4.5%)
Others	17 (12.9%)
Child-Pugh class	
A	124 (93.9%)
B	8 (6.1%)
Performance status	
ECOG 0	85 (64.4%)
ECOG 1	47 (35.6%)
BCLC stage	
A	10 (7.6%)
B	75 (56.8%)
C	47 (35.6%)
Tumor burden	
Tumor size (cm)	8.0 ± 3.7/7.1 [5.2–9.8]
No. of HCC nodules	2.1 ± 1.9/1 [1–2.25]
AFP (ng/dl)	1188.4 ± 2774.7/230.8 [8.7–7713.5]
Leukocyte (×10E9/L)	5.9 ± 2.7/5.3 [4.2–6.9]
Hemoglobin (g/L)	136.5 ± 20.6/138.0 [126.0–192.5]
Platelets (×10E9/L)	150.1 ± 89.3/134.0 [91.0–192.3]
International normalized ratio	1.16 ± 0.13/1.10 [1.02–1.17]
Alanine aminotransferase (U/L)	45.6 ± 32.3/37 [24.5–57.0]
Aspartate aminotransferase (U/L)	53.1 ± 32.7/40.5 [30–69.5]
Albumin (g/L)	39.7 ± 5.3/39.8 [36.6–42.5]
Total bilirubin (μmol/L)	16.6 ± 6.8/15.3 [11.6–19.6]
Urea nitrogen (mmol/L)	5.2 ± 1.7/4.8 [4.2–6]
Serum creatinine (umol/L)	84.2 ± 15.9/82.5 [73.8–95.0]

Abbreviations: *S.D*. standard deviation, *IQR* interquartile range, *ECOG* Eastern Cooperative Oncology Group, *BCLC* Barcelona Clinic Liver Cancer, *AFP* alpha-fetoprotein

### Hypervascularity as a favorable predictor for response, TTP and OS

**Fig. 1:**
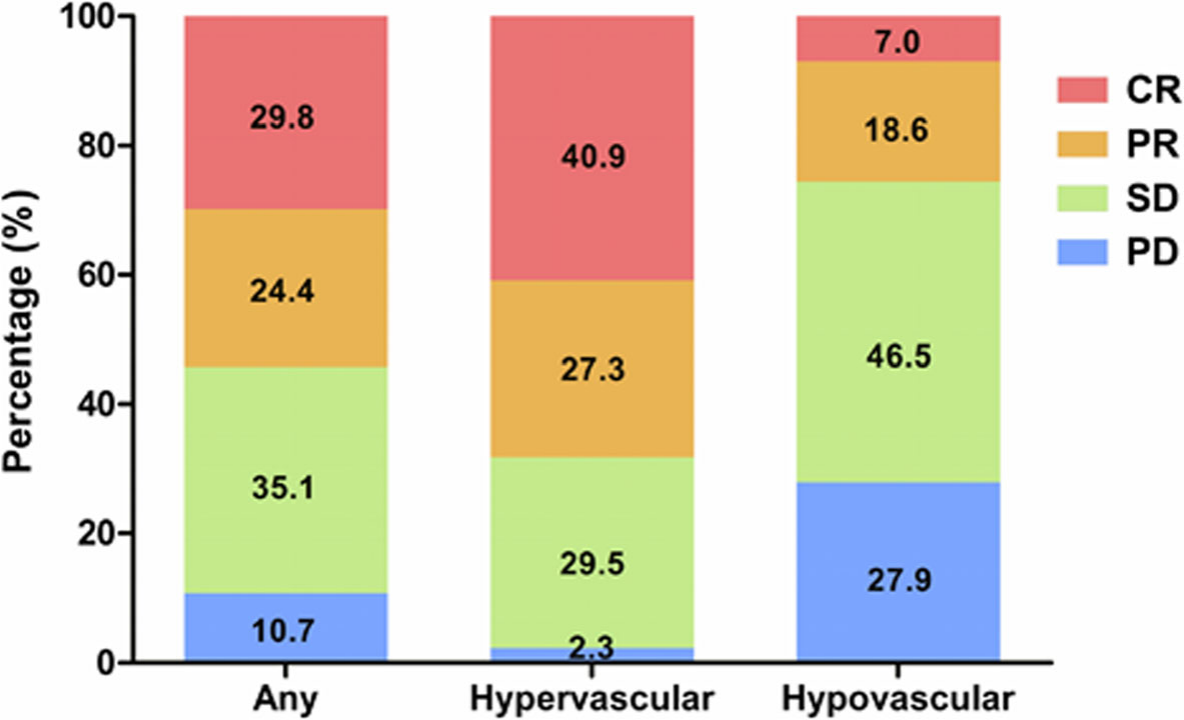
Radiologic response to the combination therapy of TACE and sorafenib, which was assessed based on the mRECIST criteria after first TACE

**Fig. 2:**
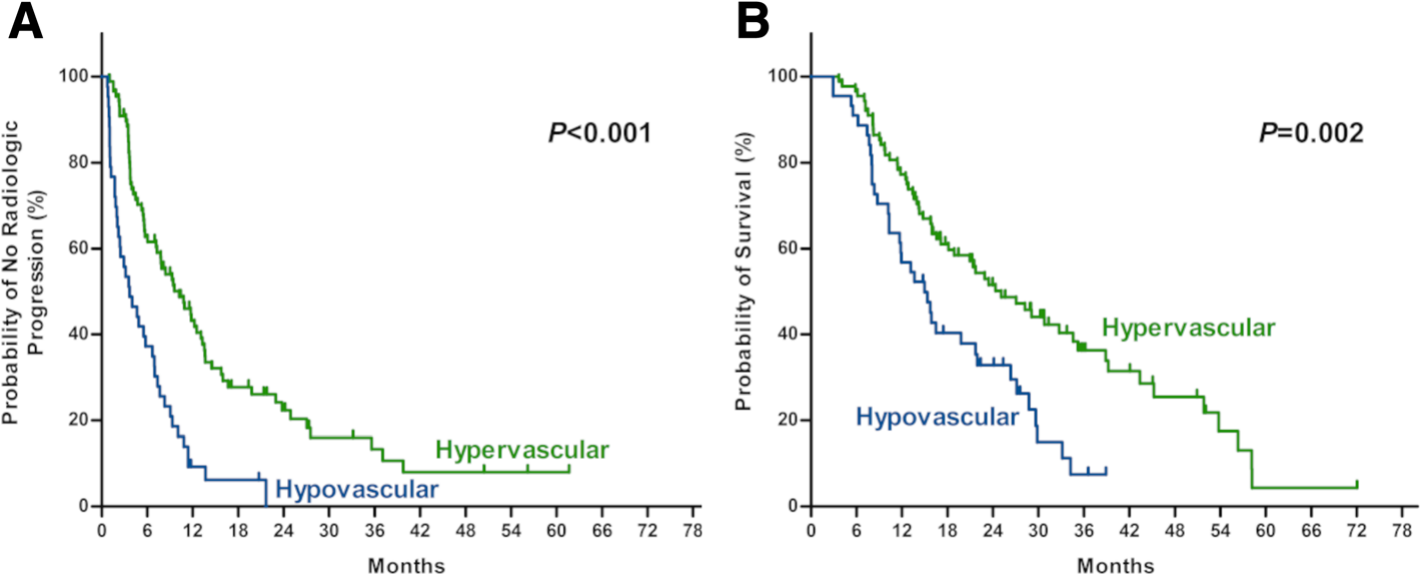
The difference in time to radiologic progression (**a**) and survival (**b**) after combination therapy of TACE and sorafenib according to vascularity in HCC patients

Among the whole cohort, tumors of 99 patients (75.0%) were stained obviously in DSA. Majority (122, 92.4%) of tumor feeding vessels were dilated and tortuous. The venous pooling and “holding ball” sign were found in 56 (42.4%) and 65 patients (49.2%), respectively. Clear tumor boundary was seen in 87 patients (65.9%), and finally 88 patients (66.7%) with homogeneous lipiodol retention were confirmed as those with hypervascularity (Additional file 1: Figure S1). In 131 patients with at least once imaging evaluation, the median time to evaluate initial response was 31 (IQR 27–35) days following first TACE treatment. Regarding the early imaging response, the complete response (CR), partial response (PR), stable disease (SD), and progression disease (PD) were in 39 (29.8%), 32 (24.4%), 46 (35.1%) and 14 (10.7%) patients, respectively, with an object response rate (CR and PR) of 54.2% (Fig. 1). Mann-Whitney analysis showed that hypervascular lesions responded better than hypovascular ones to combination treatment of TACE and sorafenib (U = 842.0, *p* < 0.001). Besides, patients with hypervascular tumors benefit more in TTP (10.2 vs 3.7 months, HR 0.38, p < 0.001) and OS (25.1 vs 15.0 month, HR 0.50, *P* = 0.002) than those with hypovascular lesions (Fig. 2 a and b).

### HFSR-response as a surrogate marker for combination treatment

**Fig. 3:**
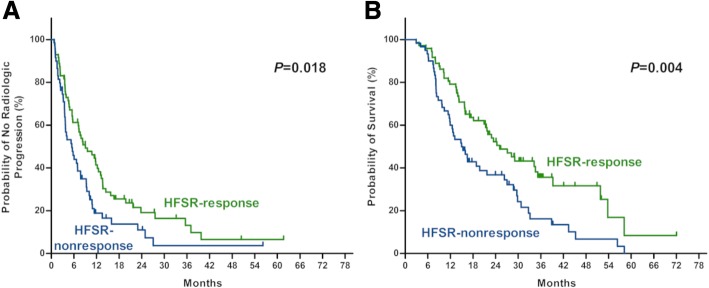
The difference in time to radiologic progression (**a**) and survival (**b**) after combination therapy of TACE and sorafenib according to HFSR-response in HCC patients

Overall, 123 patients (93.2%) presented at least one adverse event during that time. The most common sorafenib-related adverse events were HFSR (107, 81.1%), alopecia (96, 72.7%), and rash (63, 47.7%). Nevertheless, majority of them were mild (62.8%) or moderate (27.8%) (Additional file 2: Table S1). For HFSR, more than half of them were severe or moderate and considered as clinical significant HFSR. In addition, 93.5% of the clinically significant HFSR appeared within 60 days after the sorafenib initiation. According to our previous definition, the development of ≥2 grade of HFSR within 60 days of sorafenib initiation as HFSR-response were observed in 72 patients, and otherwise as HFSR-nonresponse in 60 patients. Besides, patients with HFSR-response were superior to those with HFSR-nonresponse in TTP (9.1 vs 5.4 months, HR 0.63, *p* = 0.018) and OS (25.1 vs 15.0 month, HR 0.56, *P* = 0.004) (Fig. 3 a and b).

### Combing vascularity and HFSR for the prediction of outcomes

**Fig. 4:**
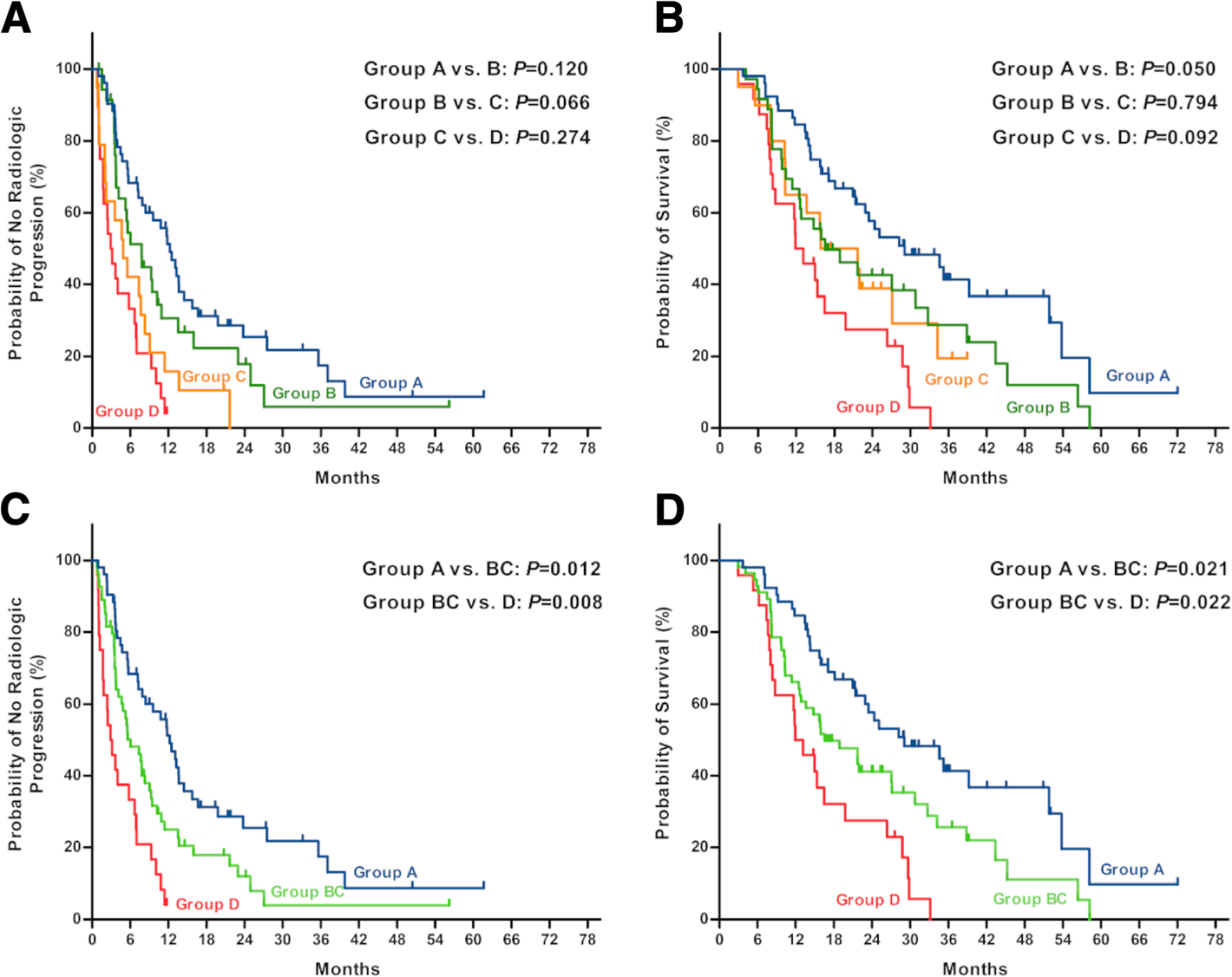
The difference in time to radiologic progression (**a**) and survival (**b**) after dividing patients into 4 groups based on vascularity and HFSR-response; the difference in time to radiologic progression (**c**) and survival (**d**) after combining group B and C into group BC (Log-rank *P* < 0.001 for comparisons of all groups at the same time). Group A: patients with both hypervascularity and HFSR-response; group B: patients with hypervascularity but HFSR-nonresponse; group C: patients with hypovascularity but HFSR-response; group D patients with hypovascularity and HFSR-nonresponse; group BC: patients with either hypervascularity or HFSR-response

Hypervascularity and the HFSR-response were favorable predictors for combination therapy, which divided patients into four distinct groups: group A included patients with both hypervascularity and HFSR-response (52 patients); group B represented patients with hypervascularity but HFSR-nonresponse (36 patients); group C included patients with hypovascularity but HFSR-response (20 patients); group D consisted of those with hypovascularity and HFSR-nonresponse (24 patients). Median TTP of group A, B, C and D were 12.2, 7.8, 4.9 and 2.9 months, respectively; and median OS of them were 29.1, 16.5, 15.9 and 11.9 months (Fig. 4 a and b). Because of the similarity in TTP and OS (*p* = 0.066 and *p* = 0.794), patients of group B and group C comprised a same stratification, group BC. Median TTP and OS of such group BC (patients with either hypervascularity or HFSR-respond) were 6.0 and 16.5 months, which were better than group D (patients with hypovascularity and HFSR-nonresponse) of 2.9 months in TTP (HR 1.99, *p* = 0.009) and 11.9 in OS (HR 1.85, *p* = 0.024). Group A of patients (with both hypervascularity and HFSR-response) achieved median TTP of 12.2 months and OS of 29.1 months, which were better than those of group BC (HR 1.74, *p* = 0.012; HR 1.73, *p* = 0.021) (Fig. 4 C and D).

### Validation and adjustment in multivariate analysis

Although the vascularity, HFSR-response, and the stratification based on them were significant predictors for TTP and OS, they hadn’t yet adjusted by other prognostic factors in multivariate analysis. Consequently, vascularity and HFSR-response, as well as the stratification, were included in four different multivariate analysis of TTP and OS (Table 2 and Table 3). The univariate analysis showed that the prognostic factors for TTP were tumor size ≥7 cm (HR 1.65, *p* = 0.011), hypovascularity (HR 2.64, *p* < 0.001) and HFSR-nonresponse (HR 1.59, *p* = 0.018), and they remained significant in multivariate analysis (HR 1.90, *p* = 0.001; HR 2.81, *p* < 0.001; HR 1.50, *p* = 0.043). Multivariate analysis for OS indicated AST ≥ 40 U/L (HR 1.86, *p* = 0.006), Child-Pugh score (HR 1.90, *p* = 0.012), multiple lesions (HR 1.75, *p* = 0.012), hypovascularity (HR 2.10, *p* = 0.001) and HFSR-nonresponse (HR 1.96, *p* = 0.002) were independent risk factors. When tumor vascularity and HFSR-response were replaced with the stratification in another two multivariate analyses, group A patients still survived group BC and group D obviously (HR 1.91, *p* = 0.010; HR 4.15, *p* < 0.001), and remained better in TTP (HR 1.87, *p* = 0.005; HR 4.11, *p* < 0.001).

**Table 2:** Prognostic factors for time to radiologic progression in HCC patients treated with combination treatment

Characteristics	NO. (N1 = 131)	mTTP (month)	Uni-variate HR (95%CI)	*p* value	Multi-variate HR1^a^ (95%CI)	*p* value	Multi-variate HR2^b^ (95%CI)	*p* value
Gender (male/female)	112/19	7.3/7.2	0.923 (0.549–1.552)	0.763				
Age (< 60/≥60 years)	88/43	7.3/7.7	1.173 (0.774–1.776)	0.451				
Etiology (HBV/other than HBV)	108/23	7.3/7.2	1.065 (0.653–1.736)	0.802				
PLT (< 150/≥150 × 10E9/L)	74/57	7.8/5.6	0.925 (0.630–1.358)	0.691				
Albumin (< 35/≥35 g/L)	20/111	12.5/6.7	0.850 (0.483–1.494)	0.571				
Bilirubin (≥17/< 17 μmol/L)	50/81	6.9/8.3	1.159 (0.782–1.720)	0.462				
AST (≥40/< 40 U/L)	68/63	5.6/7.8	1.246 (0.851–1.823)	0.258				
ALT (≥40/< 40 U/L)	55/76	5.8/7.4	1.044 (0.708–1.540)	0.827				
AFP (≥200/< 200 ng/ml)	66/65	4.9/9.3	1.174 (0.800–1.723)	0.412				
Ascites (with/without)	15/116	5.7/7.3	1.291 (0.688–2.424)	0.426				
Child-Pugh score (5/6/≥7)	103/20/8	7.0/6/7/21.7	0.919 (0.641–1.316)	0.644				
Tumor size (≥7/< 7 cm)	70/61	5.4/10.8	1.646 (1.119–2.422)	0.011	1.900 (1.282–2.815)	0.001	1.834(1.239–2.741)	0.002
No. of HCC nodules (≥2/1)	62/69	5.4/9.6	1.338 (0.911–1.966)	0.137				
Sorafenib usage (after/before TACE)	43/88	7.9/7.2	0.998 (0.670–1.488)	0.993				
Vascularity (hypovascular/hypervascular)	43/88	3.7/10.2	2.643 (1.756–3.978)	< 0.001	2.807 (1.848–4.265)	< 0.001		
HFSR (non-responder/responder)	60/71	5.4/9.1	1.594 (1.084–2.343)	0.018	1.500 (1.031–2.221)	0.043		
Stratification^c^								
Group A	52	12.2	1				1	
Group BC	55	6.0	1.742 (1.127–2.693)	0.012			1.868 (1.205–2.896)	0.005
Group D	24	2.9	3.741 (2.166–6.460)	< 0.001			4.108 (2.362–7.145)	< 0.001

^a^Multi-variate HR1, variables of vascularity and 2HFSR60 were included in Cox-regression but not stratification

^b^Multi-variate HR2, stratification was included after excepting vascularity and 2HFSR

^c^Group A: patients with both hypervascularity and HFSR-response; Group BC: patients with either hypervascularity or HFSR-response; Group D patients with hypovascularity and HFSR-nonresponse

**Table 3:** Prognostic factors for overall survival in HCC patients treated with combination treatment

Characteristics	NO. (N2 = 132)	mOS (month)	Uni-variate HR (95%CI)	*p* value	Multi-variate HR1^a^ (95%CI)	*p* value	Multi-variate HR2^b^ (95%CI)	*p* value
Gender (male/female)	112/20	21.7/15.8	1.095 (0.625–1.917)	0.752				
Age (< 60/≥60 years)	89/43	21.0/21.7	1.109 (0.720–1.708)	0.640				
Etiology (HBV/other than HBV)	109/23	21.0/26.3	1.154 (0.688–1.934)	0.587				
PLT (< 150/≥150 × 10E9/L)	74/58	21.7/21.4	0.970 (0.647–1.457)	0.885				
Albumin (< 35/≥35 g/L)	20/112	14.3/23.4	1.627 (0.959–2.763)	0.071				
Bilirubin (≥17/< 17 μmol/L)	51/81	15.9/27.1	1.573 (1.045–2.368)	0.030	1.397 (0.915–2.132)	0.121	1.371 (0.878–2.139)	0.165
AST (≥40/< 40 U/L)	69/63	14.8/32.7	2.008 (1.329–3.034)	0.001	1.859 (1.194–2.894)	0.006	1.854 (1.193–2.883)	0.006
ALT (≥40/< 40 U/L)	56/76	17.1/21.7	0.943 (0.624–1.427)	0.783				
AFP (≥200/< 200 ng/ml)	67/65	15.7/28.7	1.533 (1.017–2.310)	0.041	1.364 (0.869–2.142)	0.178	1.379 (0.884–2.152)	0.157
Ascites (with/without)	15/117	12.6/21.9	1.975 (1.093–3.569)	0.024	1.136 (0.470–2.764)	0.777	1.149 (0.477–2.769)	0.757
Child-Pugh score (5/6/≥7)	104/20/8	25.1/17.1/12.5	1.606 (1.149–2.244)	0.006	1.895 (1.150–3.123)	0.012	1.907 (1.160–3.137)	0.011
Tumor size (≥7/< 7 cm)	71/61	16.5/26.3	1.457 (0.966–2.199)	0.073				
No. of HCC nodules (≥2/1)	63/69	14.8/27.1	1.806 (1.200–2.718)	0.005	1.753 (1.134–2.709)	0.012	1.764 (1.138–2.733)	0.011
Sorafenib usage (after/before TACE)	43/89	22.9/21.0	0.932 (0.608–1.430)	0.747				
Vascularity (hypovascular/hypervascular)	44/88	15.0/25.1	2.003 (1.300–3.085)	0.002	2.103 (1.355–3.266)	0.001		
HFSR (non-responder/responder)	60/72	15.0/25.1	1.801 (1.201–2.700)	0.004	1.957 (1.281–2.991)	0.002		
Stratification								
Group A	52	29.1	1				1	
Group BC	56	16.5	1.729 (1.085–2.758)	0.021			1.910 (1.167–3.127)	0.010
Group D	24	11.9	3.166 (1.802–5.563)	< 0.001			4.154 (2.311–7.465)	< 0.001

^a^Multi-variate HR1, variables of vascularity and 2HFSR60 were included in Cox-regression but not stratification

^b^Multi-variate HR2, stratification was included after excepting vascularity and 2HFSR

^c^Group A: patients with both hypervascularity and HFSR-response; Group BC: patients with either hypervascularity or HFSR-response; Group D patients with hypovascularity and HFSR-nonresponse

## Discussion

The present study showed that tumor hypervascularity and development of ≥2 grade of HFSR within 60 days of sorafenib initiation (HFSR-response) were predictors of better outcome in 132 patients with intermediate HCC treated by combination therapy of TACE and sorafenib. Although the vascularity and HFSR have been previously regarded as predictive factors of TACE alone or sorafenib alone therapies respectively, their predictive values in combination treatment were rarely assessed to our best knowledge.

Previous studies considered tumor vascularity as a predictor of efficacy for TACE treatment; but their estimations of hypervascularity mainly depended on control-enhanced CT or MRI, which assessed tumor vascularity indirectly and inaccurately, and varied among observers [18–20]. DSA was a direct method of vascularity assessment, and the definition of hypervascularity were mostly described as “tumor stained obviously or more vascularity than nontumorous hepatic parenchyma” in previous studies [33, 34]. However, present study revealed that 75% patients had the characteristic of tumor stained obviously, 92.4% patients with tumor vessels tortuous and dilated; this might overestimate the tumor hypervascularity. Consequently, the judgment on tumor vascularity should combine vessel signs with immediate lipiodol retention, which results would correlate with efficacy of TACE better. There were 66.7% of patients with hypervascular tumors in present study, which was comparable with previous reports (59.6–95% by CT or MRI [18–20]; 71.4–92% by DSA [34, 35].

Vincenzi et al. firstly conducted a retrospective study to evaluate the role of early cutaneous toxicity as a surrogate marker of efficacy in advanced HCC patients treated with sorafenib [29]; and then its predictive value was validated in a prospective cohort of 147 HCC patients conducted by BCLC group with the land-mark analysis [26]. The predictive abilities of sorafenib related AEs for outcomes had been widely recognized, but the definition varied across different studies [21]. In addition, our previous study had established a three-dimensional criterion incorporating the type, severity and occurrence time to categorize sorafenib-related adverse events, evaluated their predictive abilities rather than merely concentrating on their correlations with treatment efficacy, found the development of a hand-foot-skin reaction (HFSR) ≥ grade 2 within 60 days after sorafenib initiation as the optimal criterion to best discriminate responders with improved survival [31]. In the present study, we defined the development of ≥2 grade of HFSR within 60 days of the sorafenib initiation as HFSR-response, which remained a significant predictor for better prognosis of combination therapy of TACE and sorafenib. It should be admitted that the used definition of HFSR-response came from our previous study which focused on the patients receiving sorafenib alone rather than combination therapy with TACE; however, in another point of view, the revealed prognostic values of HFSR in combination therapy validated and expanded our previous findings. In addition, though the occurrence of HFSR means more survival benefits, the non-occurrence of HFSR could not indicate no survival benefits for the absence of studies comparing these patients with untreated patients.

Although this study didn’t answer if the combination treatment of TACE and sorafenib was superior to TACE or to sorafenib monotherapy; it indicated that tumor hypervascularity and HFSR-response were robust predictive factors for better outcome, and patients with both characteristics survived best from the combination therapy of TACE plus sorafenib. According to previous reports, the patients with intermediate HCC and treated by combination therapy of TACE and sorafenib reached a median TTP of 5.4 to 16.4 months, median OS of 18.5 to nearly 3 years, respectively [14–16, 30, 36, 37]. For the whole cohort of present study, the median TTP and OS were 7.3 months and 21.4 months, which kept consistent with previous studies. However, for the patients with both hypervascularity and HFSR-response, median TTP and OS reached 12.2 months and 29.1 months, which were better than most of those reports in previous studies. Additionally, our study also revealed that not all patients would benefit the same from combination treatment and the stratifications based on predictive factors should be taken into consideration.

Our study had several limitations. Firstly, the single-center nature might limit its representativeness; however, the quality control was ensured because all administrations were completed by the same experienced team. Secondly, it is undeniable that the retrospective analysis might introduce some bias; yet the prospectively collected records maximized the quality of the data. Finally, all patients in our study were Chinese with HBV infection being the major etiology, thus extrapolation and generalization of our results should be cautious and future studies are needed.

In summary, for patients treated by combination treatment of TACE and sorafenib, we reported that hypervascularity and development of ≥2 grade of HFSR within 60 days of sorafenib initiation (HFSR-response) were robust predictors for better outcomes, and the patients with both might be the best candidates, which might facilitate better prognostic stratification and clinical decision making.

## Conclusions

Tumor hypervascularity and development of ≥2 grade of hand-foot-skin reaction within 60 days were favorable predictive factors for combination treatment of TACE and sorafenib, with both of which patients might be the potential candidates and survival best.

## Additional file

Additional file 1: **Figure S1.** Descriptions of vascular characteristics in 132 patients with intermediate HCC according to DSA. (TIF 2138 kb)

Additional file 2: **Table S1.** Number (percentage) of patients reporting nonlaboratory sorafenib related adverse events by CTCAE grading. (DOCX 16 kb)

## Abbreviations

AEs: Adverse events
BCLC: Barcelona clinic liver cancer
CI: Confidence interval
CT: Computed tomography
ECOG: Eastern cooperative oncology group
HCC: Hepatocellular carcinoma
HFSR: Hand-foot skin reaction
HR: Hazard ratio
mRECIST: Modified response evaluation criteria in solid tumors
OS: Overall survival
TACE: Transarterial chemoembolization
TTP: Time to progression
