# Supplementary material for: Tumor Hypervascularity and hand-foot-skin reaction predict better outcomes in combination treatment of TACE and Sorafenib for intermediate hepatocellular carcinoma
Source: BMC Cancer. 2019 Apr 30;19:409. doi: 10.1186/s12885-019-5570-z (PMC6492437; doi:10.1186/s12885-019-5570-z)
Supplement: Supplementary file 2 — Table S1. Number (percentage) of patients reporting nonlaboratory sorafenib related adverse events by CTCAE grading. (DOCX 16 kb) [file 12885_2019_5570_MOESM2_ESM.docx]

**Additional file 2: Table S1.** Number (percentage) of patients reporting nonlaboratory sorafenib related adverse events by CTCAE grading.

| Adverse Events | Any n (%) | Grade 1 n (%) | Grade 2 n (%) | Grade 3 n (%) | Grade 4 n (%) |
| --- | --- | --- | --- | --- | --- |
| HFSR | 107 (81.1) | 30 (22.7) | 51 (38.6) | 26 (19.7) | 0 (0.0) |
| Alopecia | 96 (72.7) | 87 (65.9) | 9 (6.8) | 0 (0.0) | 0 (0.0) |
| Rash | 63 (47.7) | 35 (26.5) | 23 (17.4) | 5 (3.8) | 0 (0.0) |
| Diarrhea | 54 (40.9) | 29 (22.0) | 20 (15.1) | 5 (3.8) | 0 (0.0) |
| Fatigue | 44 (33.3) | 43 (32.6) | 1 (0.7) | 0 (0.0) | 0 (0.0) |
| Voice Change | 24 (18.2) | 21 (15.9) | 3 (2.3) | 0 (0.0) | 0 (0.0) |
| Hypertension | 15 (11.4) | 8 (6.1) | 5 (3.8) | 2 (1.6) | 0 (0.0) |
| Abbreviations: CTCAE, common terminology criteria for adverse events; HFSR, hand-foot-skin reaction. | | | | | |
|  |  |  |  |  |  |
